# Life–History Traits of *Eremias pleskei* Nikolsky, 1905 from Northeastern Anatolia

**DOI:** 10.3390/ani14233373

**Published:** 2024-11-23

**Authors:** Kamil Candan, Elif Yıldırım Caynak, Serkan Gül, Yusuf Kumlutaş, Çetin Ilgaz, Cantekin Dursun

**Affiliations:** 1 Department of Biology, Faculty of Science, Dokuz Eylül University, Buca, İzmir 35390, Türkiye; kamil.candan@deu.edu.tr (K.C.); yildirim.elif@deu.edu.tr (E.Y.C.); yusuf.kumlutas@deu.edu.tr (Y.K.); cetin.ilgaz@deu.edu.tr (Ç.I.); 2 Fauna and Flora Research and Application Center, Dokuz Eylül University, Buca, İzmir 35390, Türkiye; 3 Department of Biology, Faculty of Arts and Sciences, Recep Tayyip Erdoğan University, Rize 53100, Türkiye; serkan.gul@erdogan.edu.tr

**Keywords:** body size, sexual dimorphism, growth, lizard

## Abstract

In this study, the life–history traits of *Eremias pleskei* are presented for the first time. A total of 27 samples (17 ♂♂ and 10 ♀♀) from Iğdır Province, Türkiye, were aged using skeletochronology. Student’s *t*-test was performed to compare mean differences in the variables between sexes. The relationships between age and snout–vent length (SVL) were determined through correlation. Growth patterns were estimated based on the von Bertalanffy equation. Lastly, survival rate and adult life expectancy were calculated for each sex. The mean SVL was 52.93 ± 1.28 mm in males and 46.23 ± 1.55 in females. The mean age was 4.88 ± 0.43 years and 3.20 ± 0.29 years for males and females, respectively. Correlation analysis revealed that age and SVL were highly correlated in males (r = 0.49) and females (r = 0.75). The growth coefficient (K) was found to be 0.28 ± 0.18 in females and 0.62 ± 0.25 in males. The survival rate was 57.14% in females and 75.38% in males. The adult life expectancy was calculated at 2.83 years and 4.56 years in females and males, respectively. To sum up, males were larger than females, indicating male-biased sexual size dimorphism (SDI: −0.144). In addition, males had a greater mean age and longevity (max male age: 8 yr; max female age: 5 yr).

## Simple Summary

Life–history traits such as age at maturity, longevity, growth patterns, and age–size relationships are primary sources to understand the population dynamics of a species. In lizards, short-lived species tend to reach sexual maturity earlier to produce more offspring than those that have longer lifespans, and this causes differences in demographic features. Moreover, these differences can occur even between populations of a single species, depending on temperature, food availability, and other environmental factors. In this respect, the life–history characteristics of *Eremias pleskei* are addressed for the first time in this study. The findings indicate that males have larger bodies consistent with higher growth coefficients than females. Moreover, the mean and maximum age were higher in males, as supported by greater survival rates and adult life expectancy. At a large scale, the obtained values resemble other representative species in the same genus.

## 1. Introduction

The life–history traits and population dynamics of natural populations are extremely important for the conservation of species. It is possible to understand the effect of the environment on growth by comparing both populations and species with different growth compositions. For lizards, age at maturity, longevity, and age–size ratio are the main demographic life–history characteristics that can shed light on the conservation approaches of species [1,2,3].

In short-lived species, certain characteristic features are observed, such as reaching sexual maturity at an early age, having a high reproduction rate throughout the year, and producing more offspring compared to species with longer lifespans [4,5]. Moreover, some life–history models may emerge depending on the dynamics and structure of populations. For instance, the lifespan of lizards with delayed sexual maturity is generally longer because they allocate most of their energy capacity to defense instead of reproduction, which results in low mortality rates in adults [6,7,8]. On the other hand, the differences in life–history traits may vary between distinct species and even populations of the same species, depending on temperature, food availability, and other environmental factors [9,10,11,12].

Skeletochronology is a widely used method to determine age structure and life–history traits in vertebrates, especially for amphibians and reptiles [13,14,15,16]. The method also facilitates calculating growth rates in organisms. The calculation in this method is based on counting lines of the arrest of growth (LAGs) of which one corresponds to a year appearing in cross-sections taken from the diaphysis of long bones obtained from toes [17]. The LAGs reflect seasonal changes in the growth rate, and growth appears as a wide band on the bone during the warm period of the year and as a thin line (line of stagnation) during the cold period.

The Transcaucasian Racerunner (*Eremias pleskei* NIKOLSKY, 1905) is a critically endangered species distributed in the restricted area on the borders of Armenia, Nakhichevan, Azerbaijan, northwestern Iran, and Türkiye. The habitat preference in the distribution range is associated with sandy and semi-desert areas [18]. Due to the potential extinction risk, the species has become the subject of different studies assessing taxonomical status and habitat preferences and suggesting conservation strategies [19,20,21,22]. Recently, no study has been conducted on the life–history traits of the species using the skeletochronology method. However, demographic parameters are crucial when applied for conservation purposes. Given the lack of data in the literature, we aimed to estimate the age structure, lifespan, and growth patterns of *E. pleskei* for the first time in this study. Moreover, the findings were compared with related *Eremias* species to evaluate demographic traits. Furthermore, in relation to the fact that this study concerns an endangered species, it was conducted on museum specimens. Museum materials are primary sources when investigating the age structure of extinct or critically endangered species to avoid invasive methods having harmful effects on population health [23]. In addition, where ethical or conservation concerns exist, previously collected specimens allow for the expansion of knowledge of the organism [24].

## 2. Materials and Methods

**Figure 1:**
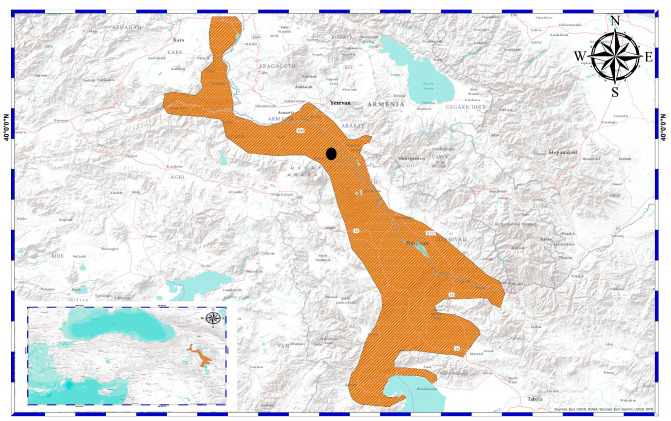
The map indicates the entire distribution range of *E. pleskei* and the black point shows the occurrence data of the sampled population in this study.

In this study, a total of 27 museum samples (17 ♂♂ and 10 ♀♀) were examined and collected from Aralık district, Iğdır Province, Türkiye, on 1 July 2002 (Figure 1). The samples were sexed following the presence of a hemipenis in the cloacal opening and secondary sexual characteristics. Body size from snout to vent (SVL) was measured using a Mitutoyo digital caliper (Ontario, Canada) to the nearest 0.01 mm. Moreover, the outermost two segments of the longest toe were clipped from the right hindlimb of each individual for age determination.

**Figure 2:**
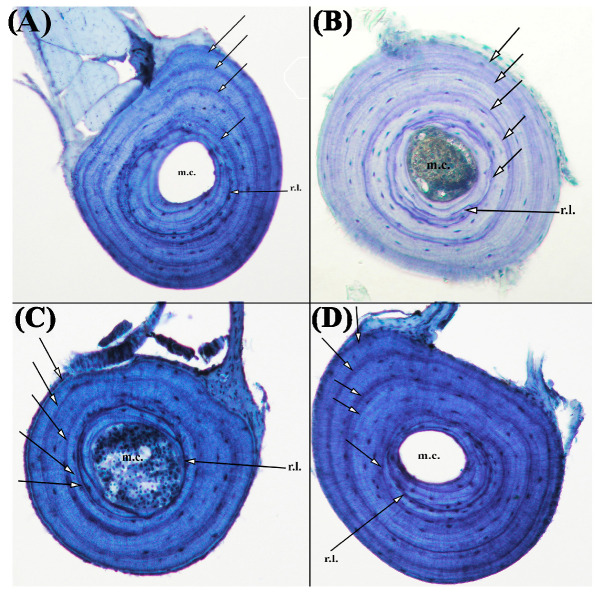
Different cross-sections obtained from the diaphysis of the phalanges of 4-year- (**A**) and 5-year (**B**–**D**)-old individuals. The innermost arrow indicates resorption line (r. l) and the other arrows demonstrate LAGs.

The standard skeletochronology method was applied to calculate age [25]. Accordingly, toe samples preserved in 70% alcohol were stripped, and then the bones were left under running tap water overnight. Subsequently, the samples were washed several times with distilled water and then placed in 5% nitric acid (HNO_3_) solution for 2.5–3 h for decalcification. After decalcification and cleaning treatment, the samples were left in xylene for 3 min and then incubated overnight in melted paraffin. Next, 16 μm bone sections were obtained from the diaphysis using a microtome where the medullary cavity was the smallest and the periosteal bone was the widest. The sections were stained with Ehrlich’s Hematoxylin for around 15 min and then fixed to slides. Photos were taken using an Olympus Camedia DP73 camera mounted on an Olympus BX53 microscope (Tokyo, Japan). Age estimation was performed by counting LAGs (Figure 2).

Descriptive statistics of all variables were calculated for each sex. The normality assumption was tested using the Kolmogorov–Smirnov test. The homogeneity of variances was checked using Levene’s test. Because the variables followed a normal distribution (*p* > 0.05) and the variances were homogenous (SVL: F_1,25_ = 0.09, *p* > 0.05; Age: F_1,25_ = 1.69, *p* > 0.05), parametric tests were used in downstream analyses. To compare mean differences between sexes, Student’s *t*-test was used. Sexual size dimorphism was assessed using the index (SDI) proposed by Lovich and Gibbons [26]: SDI = [(size of larger sex/size of smaller sex) ± 1]. To estimate relationships between age and SVL, Pearson’s correlation test and linear regression model were applied.

Growth curve models were estimated using the typical von Bertalanffy’s equation modified by Beverton and Holt [27]: Lt = L∞{1 − exp[−k(t − t_0_)]}, where Lt is the expected or average length at the time (or age) t, L∞ is the asymptotic average length, k is the so-called body growth rate coefficient, and t_0_ is a modeling artifact that is said to represent the time or age when the average length was zero. To visualize the growth curves, a hypothetical individual was added to the dataset using the reference of Kim et al. [28] under the presented parameters: SVL_0_ at hatching is fixed to a mean of 26.60 mm and t_0_ (age at hatching) is 0.15 years. Moreover, the survival rate [29] and adult life expectancy [30] of each sex were identified to reveal life–history characteristics. The models and indexes were examined following “fishR Vignette” prepared by Ogle [31]. All analyses were performed using R Programming Language v4.1.2 [32].

## 3. Results

**Figure 3:**
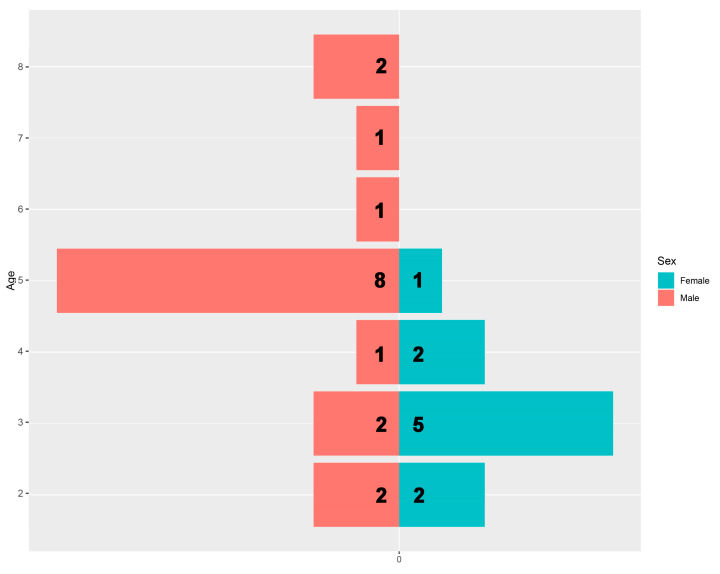
Population pyramid graphic demonstrating the age classes with the number of individuals in both sexes. Numbers on the bar plots refer to the number of individuals in each age group.

According to the summarized statistics, the mean SVL with the standard error was found to be 52.93 ± 1.28 mm in males. The minimum SVL was 38.12 mm whereas the maximum was 60.78 mm (range: 22.66 mm). The mean age was 4.88 ± 0.43 years with a minimum of 2 years and a maximum of 8 years (range: 6). For females, the mean SVL was 46.23 ± 1.55 mm, for which the minimum was 36.72 mm, and the maximum was 54.60 mm (range: 17.88 mm). The mean age was 3.20 ± 0.29 years while the minimum was 2 years and the maximum was 5 years (range: 3 years; Figure 3).

**Figure 4:**
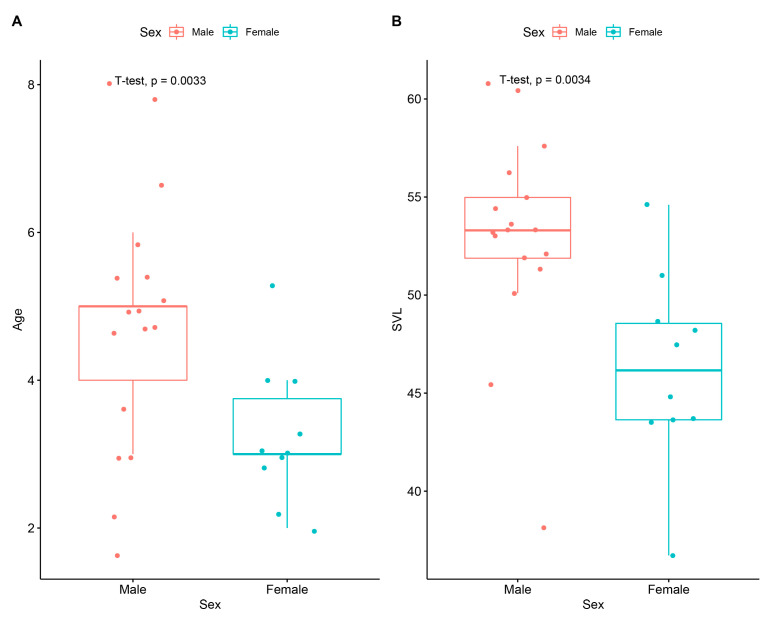
Boxplots of data resulting from the intersexual differences in age (**A**) and SVL (**B**). The tick line in the box shows the median. The lines positioned under and on the box are whiskers. The minimum and maximum values and interquartile range are represented with jitters.

Levene’s test results showed that variances were homogenous in age (F_1,25_ = 1.69; *p* > 0.05) and SVL (F_1,25_ = 0.09; *p* > 0.05). Student’s *t*-test findings indicated that the sexes were significantly different in terms of mean age (t = 3.12; df = 25; *p* < 0.01) and SVL (t = 2.78; df = 25; *p* < 0.05). The SDI index was determined to be −0.144, indicating male-biased sexual size dimorphism. The distribution of data was visualized with boxplots, as presented in Figure 4.

**Figure 5:**
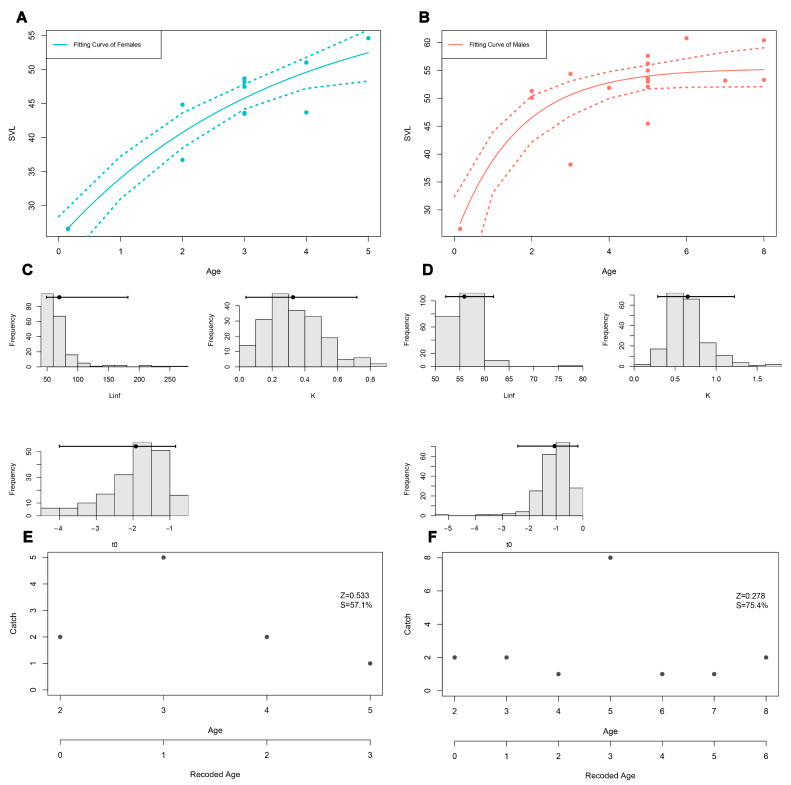
The constructed von Bertanlaffy growth curve models, parameters, and survival rate scatter plot in females (**A**,**C**,**E**) and males (**B**,**D**,**F**).

The correlation test revealed that age and SVL were highly correlated, and the direction of the relationship between variables was positive both in males (r = 0.49, *p* < 0.05) and females (r = 0.75, *p* < 0.05). The von Bertalanffy growth models adequately fitted the age and SVL relationship; however, the growth trajectories were not identical between the sexes. The growth coefficient (K) with standard error was found to be 0.28 ± 0.18 (95% CI: 0.05–0.77) in females and 0.62 ± 0.25 (95% CI: 0.27–1.41) in males. Moreover, the estimated asymptotic SVL values were 61.22 ± 13.46 mm (95% CI: 49.03 mm–151.28 mm) and 55.35 ± 2.32 mm (95% CI: 52.53 mm–61.39 mm) in females and males, respectively. The estimated SVL value was greater than the maximum measured SVL value in females but did not exceed it in males. The curve showed a clear decrease in growth rate after 3 years in males whereas it was ongoing in females (Figure 5). The percent survival rate with standard error was found to be 57.14 ± 11.06 in females and 75.38 ± 5.38 in males. The adult life expectancy was calculated to be 2.83 years and 4.56 years in females and males, respectively.

## 4. Discussion

This study presents the first data providing information on certain life–history characteristics such as the lifespan, sexual dimorphism, longevity, and age structure of *Eremias pleskei* using skeletochronology. In summarizing general trends in the studied population, males were significantly larger than females in body size as well as in the other investigated life–history parameters such as greater mean age, maximum age, and expectancy rate in lifespan.

The time of sexual maturation of *E. pleskei* was found to be 3 years in both sexes. Sexual dimorphism in *Eremias* is characterized by longer hindlimbs and shorter trunks in males compared to females [33]. Considering male-biased sexual size dimorphism in *E. pleskei*, it can be said that this difference may be caused by sexual selection. A larger body in male lacertid lizards is related to head shape. Larger heads in males are thought as an advantage in male–male competition to subdue potential mates during mating, territorial defense, and predatory advantages [34,35,36,37,38,39].

Furthermore, numerous studies have been conducted to explore body size differences between the sexes of species from the genus *Eremias*, which are summarized in Table 1. In this respect, it can be seen that sexual size dimorphism is male biased in *E. pleskei* as observed in *E. velox*, but the general trend in the genus indicates that size differences are not significant in other taxa. Moreover, *E. pleskei* females are smaller compared to other species while males are only larger than *E. argus* individuals.

For many reptiles, external sexual dimorphism is observed, which among lizards is mainly expressed in differences in body size between the sexes. There are two main trends in the strategy of evolution (sexual selection): (1) selection for larger male size (associated with male–male combat and territorial defense) [57] and (2) selection in favor of larger female size (which provides an advantage in the choice of fertility) [58].

Various authors note a tendency for increased sexual differences in island populations of reptiles, characterized by a limited area of habitats and high density. This is firstly manifested in the increase in the size and proportions of the body in males [59,60,61]. A similar trend was found in populations living in unfavorable environmental conditions [62]. At the same time, issues related to various aspects of this interesting and important biological phenomenon, including the genus *Eremias*, have not yet been sufficiently studied. The presented study complements the lack of data.

For a number of lizard species, it is noted that the role in the differences between females and males is not only in body length but also in head size [63], which has been shown in laboratory experiments in aggressive encounters and mating; males with a larger head have an advantage over males with a smaller head, for *Zootoca vivipara* [64].

In general, for lizards, a wide range of data on the magnitude and nature of the manifestation of sexual differences are noted; this can be explained by the high intra-population variability of the body length of lizards, which is determined, on the one hand, by individual differences and, on the other, interannual variations. The latter can be explained by fluctuations in weather conditions and food abundance [65]. Based on the results of the analysis, it can be concluded that a general pattern in the manifestation of sexual differences in body length in a number of the studied species may not be evident [66] when only the size of individuals is taken into account, including the size of the head, without age analysis and growth rates (speed), using precise methods, for example, skeletochronology [67]. Using skeletochronology methods will allow us to obtain a more accurate interpretation of the data, for example, taking into account lower growth rates in unfavorable conditions or the manifestation of sexual selection for body size in males.

As presented in Table 1, different lizard species can follow different sexual dimorphism trends. To sum up male-biased reports, it is thought that sexual dimorphism has a multifactorial background primarily affected by sexual selection (through male–male competition and female mate choice), as well as ecological pressures, reproductive roles, and genetic factors. While male-biased SSD is common, the exact causes can vary widely between species, depending on their specific mating systems, ecological contexts, and evolutionary histories. For instance, Jiménez-Arcos [68] focused on the interplay between natural and sexual selection to understand SSD in *Sceloporus* lizards, and they found that the impact of sexual selection is stronger than fecundity selection which leads to size differences, and they noted that 14 out of 56 species showed male-biased SSD. Butler and Losos [69] assessed the sexual dimorphic traits of 15 different *Anolis* lizards, and they recorded larger SVL values in the males of all studied species. They proposed that independent adaptation of the sexes related to ecological factors caused size differences. Valdecantos et al. [70] investigated size differences in 29 *Phymaturus* species and they revealed that the direction of SSD was changeable under the impact of selection pressures. In this respect, literature data with different species support the variation in the genus *Eremias* based on SSD.

According to the findings of this study, males ranged in age from 2 to 8 years, while females ranged in age from 2 to 5 years. The mean age values were determined to be 4.88 and 3.20 years, respectively. The maximum age in the closely related species *E. strauchi* was 5 years (mean age: 4.66 ± 0.51) for females and 7 years (mean age: 4.91 ± 0.99) for males [42]. Üzüm et al. [43] reported that *E. suphani* has a maximum age of 10 years (mean: 7.38 ± 0.22) in females and 9 years (mean: 7.86 ± 0.51) in males. In the study by Kim et al. [28], the maximum age was found to be 11 in females (mean age: 4.50 ± 0.40), while males reached up to 8 years (mean age: 3.70 ± 0.30) in *E. argus*. Considering the mean values in terms of lifespan, *E. pleskei* was similar to *E. strauchi* and *E. argus* but had lower values than *E. suphani*. Contrary to this study, the previous reports presented above did note a significant difference between the sexes in terms of age structure in those three taxa. In reptiles, it is known that age structure and life history traits can show great differences between members of a genus even compared to populations of the same species regarding altitudinal/latitudinal gradients and demographic factors [9,71,72]. Therefore, more data are required from different populations inhabiting various environments to make a comprehensive assessment of the examined species.

The relationship between age and SVL in both sexes pointed to the presence of a positive correlation, which is also noted in the closely related species *E. strauchi*, (males: r = 0.90, *p* < 0.01; females: r = 0.97, *p* < 0.01) and *E. suphani* (males: r = 0.79, *p* < 0.01; females: r = 0.96, *p* < 0.01). However, the linear relationship between variables was weaker in *E. pleskei*, and this could be related to the size differences among these taxa. Arakelyan [73] aged the parthenogenetic lizards *Darevskia sapphirina*, *D. uzzelli*, *D. armeniaca*, and *D. unisexualis*, and found that *D. sapphirina* showed different structures in cross-sections of the narrowest periosteal bone as a result of the high rate of endosteal resorption, resulting in complete destruction of the hatchling line and the line of the first hibernation. In this study, to avoid probable errors in age estimation due to medullary resorption, diaphysis sections in which the periosteal bone size reaches its maximum and that of the medullar cavity is at its minimum were used. The age–SVL relationship is a crucial parameter in demographic investigations, and it can be shaped by some distinct factors such as hatching size, age at sexual maturity, and longer growth period [74,75]. On the other hand, the same relationship has been observed in different lacertid species such as *Podarcis muralis* [8], *Phoenicolacerta laevis* [36], and *Lacerta pamphylica* [76].

The constructed von Bertalanffy growth curves yielded well-fitted models in both sexes, but growth trajectories were different between the sexes. The lower growth rate (K) in females supports that they reach asymptotic body length later than males. For *E. argus*, the growth coefficient and asymptotic SVL were 0.26 ± 0.08 and 70.10 ± 3.90 mm in males compared to 0.22 ± 0.04 and 67.80 ± 7.40 mm in females. Accordingly, *E. pleskei* grew faster compared to *E. argus* in both sexes, which may be associated with the lifespan being higher in *E. argus* (9 years in males; 11 years in females). In other respects, a higher growth coefficient in males has been observed in different lacertid lizards. Guarino et al. [9] aged a high Alpine population of *Lacerta agilis* in Italy, and the K values were calculated as 0.63 ± 0.04 in males and 0.40 ± 0.03 in females. Eroğlu et al. [77] investigated growth patterns in *Podarcis siculus*, and they noted a K value of 0.59 ± 0.31 in males compared to 0.37 ± 0.21 in females. Given these reports, it can be stated that the K value may be associated with size, and the larger sex has a higher growth coefficient than the smaller one.

As a shortcoming, the ongoing growth of females in this study may be associated with the low number of examined adult females. In the absence of enough sexually mature and well-grown females, the fit of the model could be less descriptive. However, it must be taken into consideration that the turnover of females can be high, and they usually die before attaining their final body size. In connection with this point, the issue of the determinate vs. indeterminate body growth of reptiles is crucial to understanding such patterns. Indeterminate body growth goes on during the entire life of individuals, and body size is more relevant to environmental conditions rather than genetic structure while determined body size is more affected by organismal genetic and environmental conditions that influence growth trajectories to some extent. Frýdlová et al. [78] investigated body growth in squamate reptiles including 164 species, and they reported that determinate growth is a more common and ancestral characteristic in lizards. Frýdlová et al. [79] stated that most Pleurodont lizards demonstrate determinate body growth while Acrodont lizards show an indeterminate growth pattern, allowing them to continue growing throughout their lives. Therefore, more *E. pleskei* females must be sampled in order to understand which type of growth pattern is observed in them.

## 5. Conclusions

To conclude, this study is the first study to illustrate the life–history traits of *E. pleskei* in the literature. The findings point out that the different growth patterns between the sexes are associated with size dimorphism. Moreover, the age structure was similar to members of the genus *Eremias*. Further studies can focus on populations under different environmental conditions to compare intraspecific variations. On the other hand, conservation plans must be implemented that are associated with climate change effects [21]. Therefore, more data should be obtained from different populations to increase our knowledge of organismal biology.

## Figures and Tables

**Table 1:** Summarized literature data on sexual size differences in the genus *Eremias* and some other lizards. The measurements are presented in mm (SSD: sexual size dimorphism; n.s: non-significant).

Species	Female SVL (Range)	Male SVL (Range)	SSD	Study
*E. argus*	49.20 ± 1.20 (30.90–65.80)	48.80 ± 1.20 (32.30–63.70)	n.s	[28]
*E. argus*	49.10 ± 0.80 (31.10–65.80)	49.60 ± 1.10 (32.30–64.90)	n.s	[40]
*E. argus*	55.60 ± 0.50 (49.50–63.30)	Not reported	Not reported	[41]
*E. strauchi*	60.82 ± 3.53 (55.13–63.65)	61.10 ± 4.76 (52.01–71.38)	n.s	[42]
*E. suphani*	58.85 ± 2.44 (52.32–70.86)	60.88 ± 2.61 (52.10–95.39)	n.s	[43]
*E. multiocellata*	61.30 ± 0.50 (54.10–68.80)	Not reported	n.s	[44]
*E. velox*	56.17 ± 0.04 (52.00–69.00)	61.83 ± 0.11 (55.00–74.00)	Male-biased	[45]
*E. velox*	58.40 ± 3.00 (54.00–65.00)	63.60 ± 2.90 (59.00–67.00)	Male-biased	[46]
*Ameiva ameiva*	128.80 ± 1.10	141.40 ± 1.60	Male-biased	[47]
*Cnemidophorus ocellifer*	69.40 ± 0.50	75.30 ± 0.60	Male-biased	[47]
*Cnemidophorus tigris*	86.50 ± 0.30	90.30 ± 0.20	Male-biased	[47]
*Japalura swinhonis*	47.20 ± 3.61	51.10 ± 2.90	Male-biased	[48]
*Podarcis hispanica*	47.63 ± 4.01	50.83 ± 5.10	Male-biased	[49]
*Podarcis bocagei*	53.51 ± 3.12	55.30 ± 3.55	Male-biased	[49]
*Leiolepis reevesii*	101.40 ± 0.70 (84.00–135.70)	115.11 ± 1.1 (84.00–166.10)	Male-biased	[50]
*Eutropis multifasciata*	97.26 ± 1.37 (75.20–120.10)	100.90 ± 1.09 (76.10–124.20)	Male-biased	[51]
*Trachylepis vittata*	77.83 ± 1.16 (69.13–90.72)	70.25 ± 0.62 (65.13–74.90)	Female-biased	[52]
*Zootaca vivipara*	58.69 ± 5.44 (50.03–71.40)	51.86 ± 3.13 (47.05–59.07)	Female-biased	[53]
*Takydromus wolteri*	48.70 ± 0.50 (38.70–57.50)	46.30 ± 0.40 (38.10–53.00)	Female-biased	[54]
*Abronia taeniata*	98.42 ± 0.70 (81.74–109.87)	96.10 ± 0.83 (74.64–113.99)	n.s	[55]
*Darevskia bithynica*	61.66 ± 1.45 (54.62–66.12)	63.20 ± 1.34 (55.24–69.56)	n.s	[56]

## Data Availability

Data available on request due to restrictions.
